# Plasma Ceramide and Glucosylceramide Metabolism Is Altered in Sporadic Parkinson's Disease and Associated with Cognitive Impairment: A Pilot Study

**DOI:** 10.1371/journal.pone.0073094

**Published:** 2013-09-18

**Authors:** Michelle M. Mielke, Walter Maetzler, Norman J. Haughey, Veera V. R. Bandaru, Rodolfo Savica, Christian Deuschle, Thomas Gasser, Ann-Kathrin Hauser, Susanne Gräber-Sultan, Erwin Schleicher, Daniela Berg, Inga Liepelt-Scarfone

**Affiliations:** 1 Departments of Health Science Research and Neurology, Mayo Clinic, Rochester, Minnesota, United States of America; 2 Hertie Institute for Clinical Brain Research, Center of Neurology, University of Tuebingen and DZNE, German Center for Neurodegenerative Diseases, Tuebingen, Germany; 3 Department of Neurology, Johns Hopkins University School of Medicine, Baltimore, Maryland, United States of America; 4 Department of Internal Medicine, University of Tuebingen, Tuebingen, Germany; Inserm U837, France

## Abstract

**Background:**

Mutations in the gene coding for glucocerebrosidase (GBA), which metabolizes glucosylceramide (a monohexosylceramide) into glucose and ceramide, is the most common genetic risk factor for sporadic Parkinson's disease (PD). GBA mutation carriers are more likely to have an earlier age of onset and to develop cognitive impairment and dementia. We hypothesized that plasma levels of lipids involved in ceramide metabolism would also be altered in PD non-GBA mutation carriers and associated with worse cognition.

**Methods:**

Plasma ceramide, monohexosylceramide, and lactosylceramide levels in 26 cognitively normal PD patients, 26 PD patients with cognitive impairment or dementia, and 5 cognitively normal non-PD controls were determined by LC/ESI/MS/MS.

**Results:**

Levels of all lipid species were higher in PD patients versus controls. Among PD patients, levels of ceramide C16:0, C18:0, C20:0, C22:0, and C24:1 and monohexosylceramide C16:0, C20:0 and C24:0 species were higher (all P<0.05) in those with versus without cognitive impairment.

**Conclusion:**

These results suggest that plasma ceramide and monohexosylceramide metabolism is altered in PD non-GBA mutation carriers and that higher levels are associated with worse cognition. Additional studies with larger sample sizes, including cognitively normal controls, are needed to confirm these findings.

## Introduction

The deposition of brain alpha-synuclein, a lipid-binding protein [1], [2], is the hallmark pathology of the neurodegenerative process underlying Parkinson's disease (PD). It has been suggested that perturbations in ceramide metabolism may contribute to alpha-synuclein deposition and the formation of Lewy bodies [3]. Indeed, recent evidence suggests that dysregulated ceramide metabolism may be directly linked to the oligomer formation of alpha synuclein [4] and also enhance its toxicity [5].

**Figure 1:**
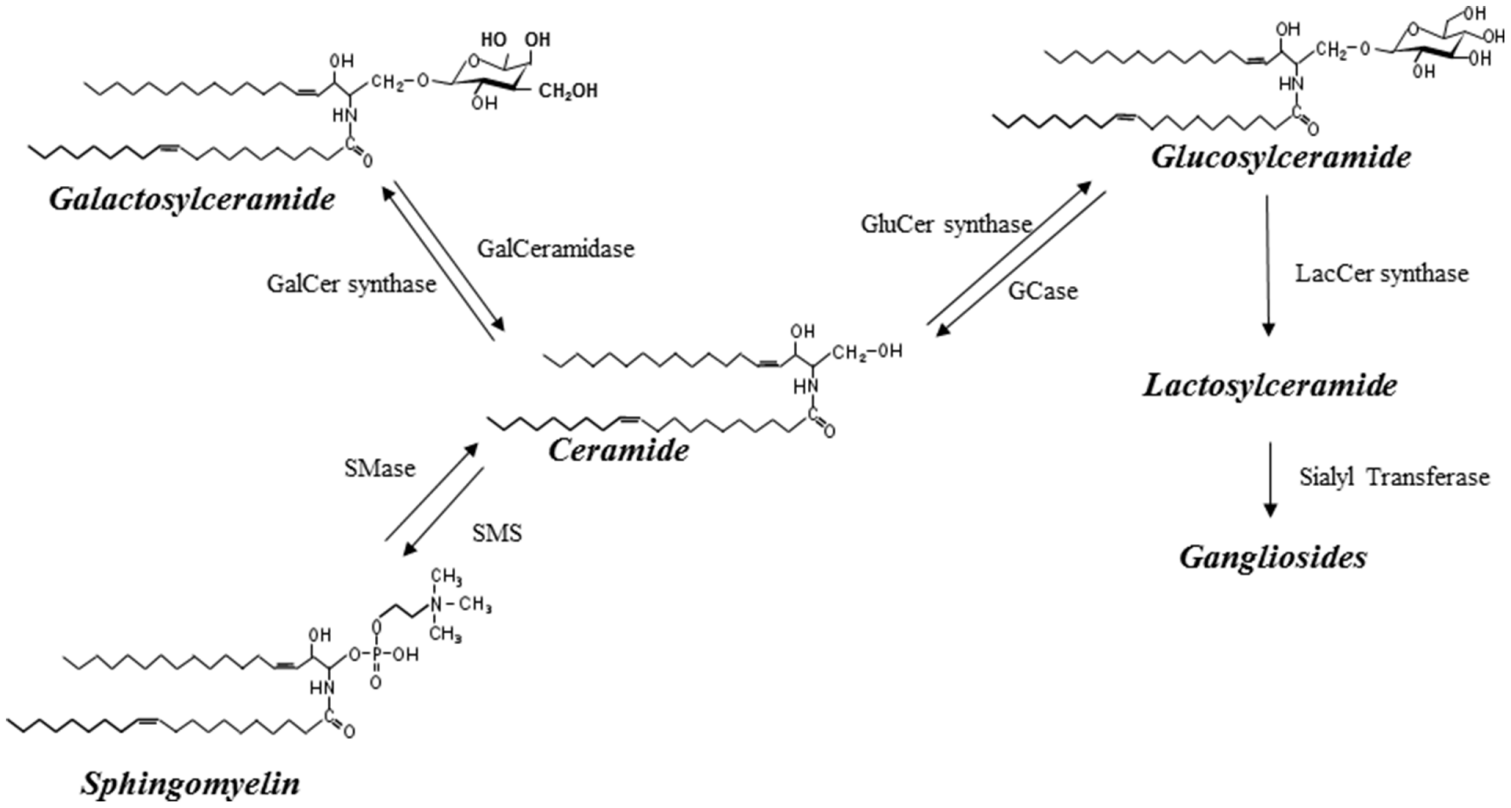
Ceramide and glycolipid metabolism. Products are indicated in bold and italics. Abbreviations for enzymes are as follows: GCase: glucocerebrosidase; GalCer synthase: galactosylceramide synthase; GluCer synthase: glucosylceramide synthase; GalCeramidase: galactosyl ceramidase; LacCer synthase: Lactosylceramide synthase; SMase: Sphingomyelinase; SMS: Sphingomyelin synthase.

Mutations in the beta-glucosidase gene (GBA) coding for glucocerebrosidase, which breaks down glucosylceramide into glucose and ceramide (Figure 1) [6], are the most common genetic risk factors for sporadic PD, comprising about 7% of all cases [7], [8]. PD patients who are GBA mutation carriers are more likely to have an earlier age of onset and to develop cognitive impairment and dementia [8], [9], [10]. These findings may suggest the critical role of glucosylceramide and ceramide metabolism in the development of PD and subsequent cognitive impairment.

To date, little research has examined the role of ceramide metabolism in PD among non-GBA mutation carriers. Indeed, it is not known whether ceramide or glucosylceramide levels are elevated in PD cases or whether higher levels are associated with cognitive impairment. The point prevalence of PD-Dementia (PDD) is close to 30% and at least 75% of PD patients who survive for more than 10 years develop dementia [11]. However, there are currently no biological or clinical biomarkers to predict those PD cases that are at greatest risk of developing cognitive impairment.

In the present study, we sought to preliminarily assess whether plasma levels of lipids involved in ceramide metabolism (ceramides, monohexosylceramide, and lactosylceramides) were altered in non-GBA mutation PD cases with and without cognitive impairment or dementia.

## Patients and Methods

### Ethics Statement

The study was approved by the ethics committee of the Medical Faculty, University of Tuebingen, Germany [12], and was performed according to the principles expressed in the Declaration of Helsinki. All participants gave their written informed consent.

### Subjects

Participants included 5 cognitively normal (CN) non-PD patients, 26 cognitively non-affected PD patients (PD-CN), 14 PD-mild cognitive impairment (PD-MCI) patients, and 12 PD-dementia (PDD) patients recruited from the ward and outpatient clinic of the Department of Neurodegenerative Diseases, Neurology Center of Tuebingen (WM, DB, TG). Eligibility criteria included a diagnosis of PD based on the UK PD Brain Bank Criteria [13], age 45–80 years, no prior surgery for PD, no diagnosis of any other neurological disease, and a MMSE score ≥18. Spouses of the patients served as controls. All controls were determined to be cognitively normal and did not have a neurological condition.

Blood was collected using standard venipuncture procedures. GBA mutations in PD patients and controls were determined by whole gene sequencing; mutation carriers were excluded. PD patients completed demographic and medical history questionnaires, had a medication inventory, and underwent a comprehensive neurological (e.g., Unified Parkinson's Disease Rating Scale, Hoehn & Yahr stage), behavioral (e.g., Apathy Evaluation Scale, short form of the Geriatric Depression Scale) and neuropsychological assessment (e.g., The Consortium to Establish a Registry for Alzheimer's Disease, Trail Making Test, Stroop Test, Brief Test of Attention, Digit Span forward and backward of the Wechsler Memory Scale revised) within the scope of the Dempark/Landscape study as previously described in detail [12].

Diagnostic criteria for PD-MCI followed the Level-1 recommendation of the Movement Disorder Society (MDS) Task Force Guidelines [14], and for PDD followed the Level-II recommendations [15].

### Sphingolipid Assays

A crude lipid extraction of plasma was conducted using a modified Bligh and Dyer procedure as previously described [16]. Analyses of sphingolipids were performed on a high-performance liquid chromatography coupled electrospray ionization tandem mass spectrometer (LC/ESI/MS/MS) (API3000s, Sciex Inc., Thornhill, Ontario, Canada) using methods similar to those previously described [16], [17].

Area under the curve was used to quantitate each sphingolipid (ceramide, monohexosylceramide, and lactosylceramide) using MultiQuant (AB Sciex). The resulting data were normalized to the corresponding internal standard. We examined several carbon-chain lengths of each sphingolipid species, rather than a sum of each total species, in order to determine whether there were chain-specific associations. Quantitation was reported as counts per second (cps). As concentrations were skewed to the right, similar to previous studies [18], [19], they were log-transformed prior to analyses.

### Statistics

Continuous variables were summarized as median (interquartile range) and categorical variables as count (percent). Wilcoxon rank sum tests were used to assess overall group differences. Mann-Whitney *U* tests were used to determine differences between all PD patients and controls, as well as between PD patients with no cognitive impairment (PD-NC) and PD patients with MCI (PD-MCI) or dementia (PDD). Spearman correlations assessed the relationships between the plasma sphingolipids and continuous variables including age, year of PD onset, Levodopa equivalent dose, body mass index, and depressive symptoms using the Geriatric Depression Scale (GDS). Logistic regression was used to assess the relationship between ceramides, monohexosylceramides and odds of cognitive impairment among PD patients, controlling for age, and GDS. All analyses were conducted using STATA Version 12.1 (StataCorp, College Station, TX).

## Results

Characteristics of the participants are shown in Table 1. The PDD and PD-NC groups were older than controls (Wilcoxon rank sum test, *P* = 0.009). However, there were no other demographic or health-related differences by group, including levodopa equivalence dose. As would be expected, performance on the PANDA, MMSE, and UPDRS declined with increasing severity of cognitive impairment (Wilcoxon rank sum test, *P* = 0.0001).

**Table 1:** Participant characteristics.

	Control (n = 5)	PD-NC (n = 26)	PD-MCI (n = 14)	PDD (n = 12)	
Characteristic	median (min, max)	median (min, max)	median (min, max)	median (min, max)	*P* value*
Age	67 (50, 75)	70.5 (45, 77)	67.5 (62, 79)	75 (68, 80)	**.009**
Male	3 (60.0%)	18 (69.2%)	11 (78.6%)	9 (75.0%)	.863
Education (years)		13.0 (8, 18)	13.0 (8, 17)	11.0 (8, 18)	.217
Body mass index	29.4 (27.3, 31.1)	26.8 (20.7, 39.1)	27.7 (21.3, 36.3)	25.0 (19.7, 34.1)	.084
Age of PD onset (years)		61.4 (43, 72)	60.3 (42, 73)	62.3 (48, 75)	.578
Geriatric Depression Scale		3.0 (0, 13)	3.0 (0, 7)	5.5 (1, 10)	.111
Total cholesterol (mg/dl)	144.5 (126.1, 266.0)	160.3 (126.0, 261.9)	156.9 (123.4, 265.2)	162.9 (112.6, 200.7)	.988
LDL cholesterol (mg/dl)	72.5 (54.2, 127.5)	86.5 (44.2, 161.1)	83.1 (63.0, 176.5)	95.1 (54.9, 115.3)	.875
HDL cholesterol (mg/dl)	53.7 (40.5, 109.9)	45.7 (31.9, 88.3)	42.4 (25.5, 75.0)	47.5 (38.1, 60.8)	.398
Triglycerides (mg/dl)	104.4 (76.2, 224.0)	97.6 (53.0, 226.7)	101.4 (40.3, 207.9)	78.3 (60.3, 196.7)	.293
Blood glucose (mg/dl)	91.3 (80.2, 147.6)	92.3 (76.7, 116.4)	106.5 (75.7, 236.1)	91.3 (62.5, 210.4)	.353
Levodopa equivalent dose (LED)		730 (52, 1960)	895 (205, 1833)	555 (310, 1090)	.387
PANDA	22.0 (14, 27)	25.0 (13, 30)	23.5 (11, 27)	10.5 (5, 18)	**.0001**
MMSE	28.0 (28, 29)	29.0 (25, 30)	28.5 (26, 30)	23.5 (20, 26)	**.0001**
UPDRS, part I		2.0 (0, 4)	3.0 (1, 6)	5.0 (3, 11)	**.0001**
UPDRS, part III	1.0 (0, 3)	21.5 (9, 40)	32 (7, 55)	36.5 (16, 48)	**.0001**

PD-NC, Parkinson's disease-Normal Cognition; PD-MCI, Parkinson's disease-Mild Cognitive Impairment; PDD, Parkinson's disease-dementia; LDL, Low density lipoprotein; HDL, high density lipoprotein; PANDA, Parkinson Neuropsychometric Dementia Assessment; MMSE, Mini-Mental State Examination; UPDRS, Unified Parkinson's Disease Rating Scale.

* *P*-values are based on Wilcoxon rank sum tests to assess overall group differences.

The sphingolipid levels did not correlate with age, age of PD onset, or Levodopa equivalence dose. They also did not differ by sex. Only monohexosylceramide C20:0 significantly inversely correlated with BMI (r = −0.417, *P* = 0.0004). Among the PD patients, several ceramides were positively correlated with depressive symptoms as measured by the GDS, including C18:0 (r = 0.41, *P* = 0.003), C20:0 (r = 0.356, *P* = 0.009), C22:0 (r = 0.300, *P* = 0.029), C22:1 (r = 0.434, *P* = 0.001), and C26:1 (r = 0.271, *P* = 0.049). Monohexosylceramides C18:0 (r = 0.291, *P* = 0.035) and C20:0 (r = 0.278, *P* = 0.044) also positively correlated with GDS score.

Compared to controls, PD patients had significantly higher (*P*<0.05) levels of nearly all ceramide, monohexosylceramide, and lactosylceramide species compared to controls (Table 2). We next determined whether these plasma sphingolipids varied by cognitive status among PD patients. As there were no differences between the PD-MCI and PDD groups in any lipid (all *P*>0.20), we combined these two groups and compared them to the PD-NC group. The cognitively impaired PD group had significantly higher levels of ceramide C16:0, C18:0, C20:0, C22:0, and C24:1 and monohexosylceramide C16:0, C20:0, and C24:0 species (all *P*<0.05; Table 2).

**Table 2:** Group differences in plasma sphingolipids involved in glucosylceramide metabolism.

Plasma log lipid	Control (n = 5) median (range)	PD-NC (n = 26) median (range)	PD-MCI (n = 14) median (range)	PDD (n = 12) median (range)	All PD vs. control *P* value	PD-NC vs. PD-MCI/PDD *P* value
**Ceramide**						
C16:0	11.00 (10.78, 11.24)	11.48 (10.93, 12.26)	11.66 (11.27, 11.95)	11.67 (11.39, 12.79)	**.0006**	**.035**
C18:0	10.76 (10.43, 11.27)	10.98 (10.24, 12.24)	11.21 (10.44, 11.77)	11.17 (10.93, 12.54)	.067	**.016**
C20:0	11.48 (11.23, 12.40)	12.21 (11.32, 13.58)	12.50 (11.80, 13.27)	12.54 (11.99, 14.10)	**.006**	**.037**
C22:0	13.00 (12.86, 13.69)	13.63 (12.83, 14.77)	13.95 (12.67, 15.09)	13.90 (13.47, 16.14)	**.010**	**.037**
C24:0	15.40 (14.69, 15.92)	15.73 (14.37, 16.85)	15.89 (14.17, 17.07)	16.03 (14.55, 18.00)	.096	.621
C26:0	11.57 (11.18, 12.19)	11.94 (9.68, 12.84)	12.16 (10.61, 13.51)	12.08 (9.70, 14.07)	.096	.510
C22:1	9.21 (9.01, 10.10)	9.86 (8.83, 10.60)	9.97 (9.51, 10.78)	9.91 (9.43, 11.92)	.051	.442
C24:1	12.50 (12.49, 13.29)	13.23 (12.34, 14.83)	13.57 (12.67, 14.40)	13.37 (13.22, 15.32)	**.007**	**.048**
C26:1	9.19 (8.92, 9.59)	9.70 (8.46, 11.05)	9.87 (8.92, 10.74)	9.91 (8.87, 11.77)	**.024**	.380
**Monohexylceramides (Glucosylceramides and Galactosylceramides)** *						
C16:0	11.21 (10.97, 11.67)	11.92 (11.03, 12.79)	12.09 (11.36, 12.78)	12.14 (11.52, 14.29)	**.001**	**.046**
C18:0	8.62 (8.45, 9.04)	9.11 (8.40, 10.15)	9.20 (8.33, 9.92)	9.15 (8.90, 10.80)	**.011**	.242
C20:0	10.19 (9.90, 10.26)	10.48 (9.79, 11.31)	10.69 (10.07, 11.33)	10.73 (10.10, 12.11)	**.010**	**.039**
C22:0	13.16 (12.74, 13.36)	13.75 (12.97, 14.61)	13.88 (13.18, 14.68)	14.03 (13.30, 15.52)	**.001**	.148
C24:0	13.64 (13.22, 13.89)	14.17 (13.15, 15.49)	14.56 (13.26, 15.29)	14.53 (13.57, 16.32)	**.006**	**.040**
C26:0	9.74 (9.37, 10.19)	10.20 (8.82, 11.16)	10.14 (8.41, 11.23)	10.35 (8.17, 12.28)	.215	.840
C16:1	9.15 (8.58, 9.50)	9.50 (8.67, 9.99)	9.73 (9.12, 10.34)	9.49 (9.21, 11.72)	**.011**	.089
C22:1	9.41 (9.11, 10.26)	9.94 (9.02, 10.59)	9.96 (9.00, 10.70)	10.02 (9.43, 11.88)	.176	.370
C24:1	9.69 (9.30, 13.30)	10.02 (8.48, 11.06)	10.03 (9.26, 11.30)	10.15 (9.27, 12.48)	.573	.272
**Lactosylceramides**						
C16:0	13.40 (13.26, 13.51)	13.72 (12.86, 14.59)	13.53 (13.40, 14.17)	13.76 (13.29, 15.16)	**.006**	.164
C18:0	8.75 (8.37, 8.95)	9.03 (8.18, 9.70)	9.24 (8.76, 9.65)	9.24 (8.59, 10.35)	**.006**	.142
C22:0	11.03 (10.35, 11.13)	11.40 (10.84, 12.25)	11.53 (11.13, 12.19)	11.64 (10.89, 12.74)	**.001**	.220
C24:0	10.61 (10.07, 10.76)	10.95 (10.44, 12.14)	11.16 (10.41, 12.05)	11.13 (10.40, 12.54)	**.009**	.621
C24:1	12.10 (11.70, 12.25)	12.57 (11.38, 13.40)	12.72 (12.09, 13.15)	12.71 (11.94, 13.84)	**.004**	.342

PD-NC, Parkinson's disease-Normal Cognition; PD-MCI, Parkinson's disease-Mild Cognitive Impairment; PDD, Parkinson's disease-dementia.

* Glucosylceramides and Galactosylceramides are isomers – this variable includes the total sum of both compounds.

In additional analyses among PD patients, we ran logistic regression models to examine the odds of cognitive impairment per unit increase in log lipids, controlling for age and depressive symptoms on the GDS. While the sample size was small, increasing ceramides C16:0 (OR 7.81, *P* = 0.084), C18:0 (OR 5.56, *P* = 0.035), and C22:0 (OR 2.47, *P* = 0.082) and monohexosylceramides C16:0 (OR 3.92, *P* = 0.097), C20:0 (OR 4.43, *P* = 0.068), C24:0 (OR 2.40, *P* = 0.079) and C16:1 (OR 7.45, *P* = 0.042) remained significantly or borderline-significantly associated with increases odds of being cognitively impaired (e.g., having a diagnosis of MCI or dementia).

We also examined other plasma lipids to determine whether the findings presented above were due to global lipid alterations or were specific to sphingolipids. Plasma total, LDL, and HDL cholesterol and triglyceride levels did not significantly differ between groups or between PD patients and controls (Table 1). These data suggest that results were specific to ceramides, monohexosylceramides, and lactosylceramides and are not due to global lipid effects.

## Discussion

In the present study, we determined whether plasma sphingolipids differed in PD patients versus controls and whether these lipid levels varied by cognitive impairment among PD patients. Importantly, we found that several species of ceramides, monohexosylceramides, and lactosylceramides were elevated in PD patients versus controls and were highest among those PD patients who had cognitive impairment (either MCI or dementia). These preliminary results suggest the importance of ceramide and monohexosylceramide metabolism in the pathophysiology of sporadic PD and could indicate a novel means of identifying individuals at increased risk of cognitive impairment.

Glucocerebrosidase catalyzes the conversion of glucosylceramide to ceramide and glucose inside lysosomes; lysosomal processing is the main degradation pathway of alpha-synuclein [20]. Mutations in the GBA gene coding for glucocerebrosidase result in a build-up of glucosylceramide (a monohexosylceramide) within the lysosome and, thus, decrease the degradation of alpha-synuclein. Notably, a recent study suggested a positive feedforward loop between increased glucosylceramide and alpha-synuclein accumulation, leading to neurodegeneration [4]. These experimental results fit with research showing that mutations in glucocerebrosidase are associated with an increased risk of sporadic PD, and that these patients have earlier ages of onset and more cognitive impairment [7], [9]. However, mutations in glucocerebrosidase, while the most common, only comprise 7% of all sporadic PD cases. Thus, mechanisms related to the underlying PD pathology in non-GBA mutation carriers remain to be identified.

We hypothesized that variations in lipids involved in ceramide metabolism, including both glucosylceramide and ceramide, might also be perturbed in persons with PD without specific GBA mutations, particularly in those with cognitive impairment. Cellular studies suggest that ceramides may be involved in the pathogenesis of PD. Dopaminergic neurons positively regulate neutral sphingomyelinase activity, thereby increasing ceramide levels, in response to oxidative stress [21]. Post-mortem and *in-vitro* PD studies have indirectly demonstrated that the activation of ceramide signaling may mediate the apoptosis observed in the substantia nigra [22]; decreasing levels of ceramide protect against MPTP, a specific dopaminergic toxin [23]. Ceramide has also been shown to induce apoptosis in dopaminergic models [24]; this effect is neutralized by the non-pathological gene products of parkin [25] and alpha-synuclein [26]. Both of these proteins have been implicated in PD and several other neurodegenerative diseases. However, little research has attempted to translate these findings to humans. In this study, we did find differences in median levels of plasma ceramides, monohexosylceramides, and lactosylceramides by PD status, and by presence of cognitive impairment among PD cases. Notably, this was not due to a global lipid effect as there were no differences in cholesterol or triglyceride levels. Thus, it is likely that the ceramide pathway is significantly altered in the disease process. We speculate that higher levels of these lipids may be a marker of more diffuse and severe alpha-synuclein deposition in the brain, leading to PD with cognitive impairment or dementia. Thus, it is possible that plasma levels of these lipids could be promising markers for the identification of different clinical PD phenotypes and as predictors of disease progression.

Among PD patients, we also found significant positive correlations between multiple species of ceramides and monohexosylceramides and depressive symptoms, measured by the GDS. One study of PD patients reported that GBA mutation carriers had higher depressive symptoms compared to non-carriers [10], but another study did not find a difference [27]. We have previously reported elevated plasma ceramide levels in individuals with a diagnosis of major depression compared to subjects without [28]. Thus, it is possible that plasma ceramides could be an indicator or predictor of depression in PD and merits further investigation.

Limitations of the study warrant consideration. First is the small sample size, especially of control subjects. While we had highly significant results despite the small sample size, replication is needed in larger studies to confirm the findings. Second, the mass spectrometry method captured monohexosylceramides and did not isolate glucosylceramides from galactosylceramides. These two lipids are isomers and require more time and extensive techniques to separate. As this was the first preliminary examination of these plasma lipids in PD patients, the more extensive techniques were not conducted. While we would hypothesize that the finding with monohexosylceramides is driven by alterations in the levels of glucosylceramides, and not galactosylceramides, future studies will need to quantify plasma levels of each lipid.

In conclusion, the present results suggest that plasma ceramide metabolism is perturbed in PD, and may be particularly important in the development of cognitive impairment among PD patients. While larger studies with longitudinal follow-up are needed to replicate the cross-sectional results and determine the lipids' predictive value, these preliminary results could indicate a novel means of identifying individuals at increased risk of PD and those patients with parkinsonism who are at risk of progressive cognitive impairment.
